# HOXA3 accelerates wound healing in diabetic and aged non-diabetic mammals

**DOI:** 10.1038/s41598-023-36933-4

**Published:** 2023-06-19

**Authors:** K. Parella, K. Moody, D. Wortel, H. Colegrove, J. A. Elser

**Affiliations:** 1 Ichor Life Sciences Inc, Lafayette, USA; 2 Ship of Theseus LLC, Philadelphia, USA; 3 Clarkson University, Potsdam, USA

**Keywords:** Ageing, Nucleic-acid therapeutics, Regenerative medicine, Geriatrics, Metabolic disorders, Skin diseases

## Abstract

Chronic wounds are characterized by a persistent, hyper-inflammatory environment that prevents progression to regenerative wound closure. Such chronic wounds are especially common in diabetic patients, often requiring distal limb amputation, but occur in non-diabetic, elderly patients as well. Induced expression of HoxA3, a member of the Homeobox family of body patterning and master regulatory transcription factors, has been shown to accelerate wound closure in diabetic mice when applied topically as a plasmid encased in a hydrogel. We now provide independent replication of those foundational in vivo diabetic wound closure studies, observing 16% faster healing (3.3 mm wounds vs 3.9 mm wounds at Day 9 post original injury of 6 mm diameter) under treatment with observable microscopic benefits. We then expand upon these findings with minimal dose threshold estimation of 1 μg HoxA3 plasmid delivered topically at a weekly interval. Furthermore, we observed similarities in natural wound healing rates between aged non-diabetic mice and young diabetic mice, which provided motivation to test topical HoxA3 plasmid in aged non-diabetic mice. We observed that HoxA3 treatment achieved complete wound closure (0 mm diameter) at 2 weeks whereas untreated wounds were only 50% closed (3 mm wound diameter). We did not observe any gross adverse effects macroscopically or via histology in these short studies. Whether as a plasmid or future alternative modality, topical HoxA3 is an attractive translational candidate for chronic wounds.

## Introduction

Diabetic foot ulcers (DFUs) are a common and devastating complication of diabetes that may take weeks to several months to heal due to impaired wound healing. As of 2018, 10.5% of the US population has diabetes^1^. Between 1 and 4% of the U.S. diabetic population experiences a DFU in a given year^2^, a statistic that has not changed in the past 2 decades^3^. Among DFUs, 14–24% will require lower limb amputation^4–6^. Therefore, about 80,000 US DFU amputations per year occur in the U.S.^7^. In addition to the decreased quality of life, per amputation hospitalization costs are $12–16 k in the US^8^.

Treatments for DFUs span many modalities, yet efficacy is difficult to assess due to small study sizes and a lack of head-to-head comparisons. As evidenced by the existing progression to amputation rate, DFU treatment is still insufficient^9^. The primary therapies still revolve around basic wound care, such as off-loading of shear stress and plantar pressure, cleaning and debridement of all devitalized tissue and callus, clearance of active infections via antiseptics, and bandaging to manage moisture and exudate^10^. As diabetes is considered to be the root causative agent of delayed wound healing, and observed elevated glycosylated hemoglobin levels are associated with 2–4 fold higher amputation risk^11^, strict glycemic control is preferred. Dressings comprised of biological materials such as human placental tissue, human amnion and chorion, umbilical cord membranes, and even bovine collagen and shark cartilage are commercially available and generally achieve 30–70% closure at 12 weeks whereas controls range from 20 to 30%^12–15^. Although one amniotic tissue graft marketed as Epifix did close 31 out of 32 wounds at 12 weeks, whereas controls closed only 18 of 35 in a 2016 clinical trial^16^. These treatments require human or animal tissue. Biologically-defined active treatments include rhEGF or PDGF growth factors embedded in topical patches, but the results merely halve the 12 week failure rate, akin to most human tissue grafts^17–19^.

Normal healing progresses through an overlapping sequence of phases starting with clotting, followed by inflammation initiated by neutrophils and macrophages to control introduced pathogens, re-epithelialization by keratinocytes, subepithelial angiogenesis, wound contracture, and scar remodeling of interim extracellular matrix^20,21^. Multiple factors contribute to impaired wound healing in DFUs, including decreases in growth factor production, angiogenesis, macrophage regenerative function, and keratinocyte and fibroblast migration and proliferation^22–26^. Specifically, the milieu of wounds must transition from an initial inflammatory phase to a regenerative phase for healing to progress. However, in the diabetic environment, wounds often persist in inflammatory state, which is notably evident in the sustained polarization of local macrophages towards their inflammatory state (M1) versus regenerative state (M2)^27^.

Homeobox protein A3 (HOXA3) is a transcription factor that promotes diabetic wound healing^28–34^. Mace et al. delivered HOXA3 expression plasmids to wounds in young Leprdb/Leprdb (db/db) diabetic mice using a methylcellulose topical gene delivery patch. The application of HOXA3 expression plasmids significantly improved wound closure rates in db/db mice compared to vehicle plasmid as controls. In a large diameter wound model (2.5 cm), HOXA3 treatment improved wound closure rates detectably as early as 7 days, and incited closure of all wounds by 42 days versus 77 for untreated mice^28^. Further rigorous in vitro^29^ and in vivo studies^30^ demonstrated enhanced neovascularization, keratinocyte migration, and endothelial cell invasion and migration compared to negative vehicle control. The mechanisms underlying the wound healing ability of HOXA3 has also been preliminarily investigated. Al Sadoun et al. showed that macrophages transduced with HOXA3 promoted M2 polarization via regulation of Pu.1/Spi1 and Stat6 compared to negative control mCherry transduction^30^. Furthermore, Mace et al. demonstrated HOXA3 treatment mobilized and recruited endothelial progenitor cells while attenuating inflammatory pathways when compared to control mice and Mahdipour et al. demonstrated HOXA3 promotes the differentiation of hematopoietic progenitor cells into proangiogenic Gr-1 + CD11b+ myeloid cells that stimulate neovascularization^29,31^. Together, these published in vitro and in vivo data provide rigorous evidence that HOXA3 can promote angiogenesis, re-epithelialization, and pro-healing macrophage polarization. Therefore, HOXA3 influences the entire wound healing program and accelerates diabetic wound healing in vivo. In the current study, we expanded upon these findings to explore dose dependencies in diabetic wounds and whether HOXA3 could also accelerate healing in aged mice, which are also subject to poor wound repair.

## Methods

All methods are reported in accordance with ARRIVE guidelines" (https://arriveguidelines.org).

### Diabetic mice

Male B6.Cg-m+/+Leprdb/J mice aged 8–12 weeks at start of study were obtained from Jackson Laboratories (Strain Code 000697). Mice were singly-housed and acclimated on-site for approximately 1  week prior to study activities. Mice were housed on a 12-h light–dark cycle at 20–24 °C (68–74 F) and 30–70% humidity. Mice were fed water and diet ad libitum throughout the study. Mice were randomized into 2 treatment groups based on blood glucose values prior to surgical wounding.

### Aged mice

Male and female C57BL/6J mice (77 weeks) were obtained from Jackson Laboratories. Mice were independently housed and acclimated on-site for approximately 1 week prior to study activities. Mice were housed as described above. Mice were randomized into 2 treatment groups prior to surgical wounding.

### Injury

All mice received two full-thickness (through the panniculus) 6 mm wounds over the shoulders using a punch. These wounds were splinted with silicone splints, non-absorbable suture, and surgical glue to prevent skin contracture. Sutures on splits were replaced as needed and care was taken not to disrupt the wounds during this replacement. Animals received once-daily treatment with a non-steroidal anti-inflammatory for 3 days following wounding.

### Plasmid construction

HOXA3-containing DH5α *E.coli* cells (Cat 18265017, Thermo) were grown for 18 h at 37 °C in Terrific Broth. The HOXA3 plasmid was then purified using a modified Zymo plasmid purification protocol with the ZymoPURE II Plasmid Gigaprep Kit (Cat # D4204. Plasmids were extracted and purified from cells per the vendor protocol. DNA was then syringe-filtered in the sterile hood using sterile 0.2 µm cellulose acetate filters. The concentration and purity of the DNA were quantified via UV-spectrometry at wavelengths of 260, 280, and 230 nm. The sequence is provided in Supplemental Figure S1.

### Plasmid expression confirmation

Human Dermal Microvascular Endothelial Cells (hDMECs) were transfected with either HOXA3 plasmid or green fluorescent protein (GFP) plasmids using the Lipofectamine 3000 kit from Thermo Fisher, following the vendor's protocol. After a 48-h incubation period post-transfection, the cells were examined for protein expression. To determine the expression of exogenous HOXA3, immunocytochemistry was performed. Immunostaining was conducted using an anti-myc tag antibody, this antibody was followed by a Cy2-conjugated secondary antibody to enable visualization of the immunostained cells. Expression of GFP in GFP transfected cells was assessed by directly measuring green fluorescence, without undergoing immunocytochemistry. Green fluorescence quantifications and corresponding images were acquired with an IncyCyte S3 imaging cytometer. Fluorescent signals of transfected cells were compared to counterparts which were treated with lipofectamine only. Error bars represent S.E.M, n = 4. Scale bars on images represent 100 µm.

### DMEC monolayer motility

Human Dermal Microvascular Endothelial Cells (hDMECs) were transfected with either HOXA3 plasmid or green fluorescent protein (GFP) plasmids using the Lipofectamine 3000 kit from Thermo Fisher, following the vendor's protocol. Following transfection, the cells were seeded in 96-well plates to form a confluent monolayer. A 600 μm scratch defect was created in the monolayer using a p200 pipette tip, and defect closure was monitored over time. Images of the scratched area were captured at regular intervals of 4 h. To quantify the rate of defect closure, the diameter of the defect was measured at two separate locations within each well. These measurements were then averaged to obtain a single value representing the defect size for each well. This approach allowed for a comprehensive assessment of the overall defect closure dynamics in the DMEC cells.

### Treatment

Methylcellulose wafers were produced by mixing 25 μg, or lower where indicated, HoxA3 plasmid DNA in 1% methylcellulose and spotted in 50 μL droplets onto parafilm using a positive-displacement pipette. Sample video of wafer formation is provided in the Supplemental Data. Wafers were allowed to air dry a maximum of 5 h prior to application, allowing them to reach a solid consistency. Plasmid wafers were prepared just prior to treatment. In most experiments, mice received sequential re-dosing by removing remnant plasmid and replacement with fresh plasmid wafers periodically. Wounds were not bandaged as the patches tended to adhere to the bandages (in pilot trials); rather, the wound surfaces were damp enough to encourage the wafers to stick directly to the damaged skin. In the rare cases where patches fell off spontaneously, they were replaced during the frequent measurements.

### Wound size measurements

Wounds were measured using digital calipers under isoflurane anesthesia. Measurements of each wound diameter were taken at four orientations (0°, 45°, 90°, and 135° where 0° is head-to-tail orientation, diagrammed in Supplemental Figure S2) and averaged for a single animal. Scabs were removed for measurement, but wounds were not debrided.

### Histology

Wounds and surrounding area of roughly 1 cm were excised from the mice and fixed in 10% neutral buffered formalin for 48 h. The wounds were then dehydrated by submerging in the following solutions: 70% ethanol (2 changes 1 h each), 85% ethanol (2 changes 1 h each), 95% ethanol (2 changes 1 h each), 100% ethanol (2 changes 1 h each), Histoclear xylene substitute for 3 h, and 3 h of liquid paraffin (3 total changes 1 h each). The tissues were placed in molds with the liquid paraffin wax, which was allowed to solidify on ice. Paraffin blocks were stored at room temperature until sectioning. Tissues were sectioned at a thickness of 5 µm, in the horizontal plane in the direction of dorsal to ventral. Once tissues were mounted on microscope slides, samples were placed on a 38 °C slide warmer until moisture disappeared. The paraffin wax was melted at 65 °C for 20 min prior to deparaffinization and subsequent staining.

### Animal handling

The primary studies were reviewed and approved by the Institutional Animal Care and Use Committees (IACUC) of Ichor Life Sciences, (OLAW Assurance No. D20-01099). Euthanasia was performed with CO_2_ overdose followed by confirmation with cervical dislocation or bilateral pneumothorax as a secondary measure. The CO_2_ was delivered using a Quietek automated euthanasia device displacing 70% of the cage volume/minute. Once the cage was filled with CO_2_, mice remained immersed for a minimum of 1 min prior to the secondary measures. Within this study, animals were handled and used strictly according to the guidelines of the American Veterinary Medical Association (AVMA).

### Mason’s trichome

Slides were incubated with 55 °C Bouin’s solution for 60 min immediately after slides were deparaffinized and rehydrated with distilled water. The slides were allowed to cool for 10 min after the 60-min incubation with Bouin’s solution. After equilibrating to room temperature, the tissues were rinsed with water until completely clear. Slides were then incubated with hematoxylin for 5 min, then immediately rinsed with tap water for 2 min. Slides were then incubated with a Biebrich Scarlet/Acid Fuchsin solution for 15 min, then immediately washed with tap water. Slides were then incubated with a phosphomolybdic/phosphotungstic acid solution for 15 min. Without washing, slides were incubated with an aniline blue solution for 10 min, then washed with deionized water. Slides were then incubated with 1% acetic acid solution for 5 min. After the incubation with acetic acid, slides were quickly cleared in 2 changes of 95% ethanol, followed by a quick exchange into xylene substitute. Slides were then mounted with toluene mounting media.

### H + E staining

Slides were deparaffinized with two 5-min incubations of histoclear (xylene substitute) followed by two changes of 100% ethanol and two changes of 95% ethanol (3 min and 2 min respectively). Slides were then rehydrated with tap water for 1 min. Slides were then stained with hematoxylin for 2 min followed by two separate 45 s rinses with tap water. Slides were then subjected to bluing reagent for 15 s. After bluing reagent, slides were washed twice with tap water for 30 s. Slides were then washed in 100% ethanol for 10 s, followed by a 3-min incubation with Eosin Y for 3 min. After incubating with Eosin Y, slides were dehydrated with 4 changes of 100% ethanol. Slides were then cleared with two consecutive 3-min incubations with histoclear. The slides were then mounted with toluene mounting media.

### Histopathology scoring

Anonymized samples (from the 9-day wounding study) were scored by a third-party board-certified pathologist. Criteria for pathological scoring of histology slides are as follows: vascular density, the number of vessels which contain visible blood cells and are lined by endothelium; max epithelial thickness, number of cell layers of the epidermis in the thickest region of epidermis visible on the slide; fibroblast cell density, determined by cell density and surrounding extra-cellular matrix gauged with Mason’ trichrome, granulation tissue formation, and presence of newly formed blood vessels. Note that a single epithelial layer is sufficient for the wound to qualify as epithelialized, however, an epithelialized wound may still be considered macroscopically open (as measured by digital calipers) due to the visible damaged tissue beneath the epithelial layer. Overall inflammation was also assessed. The numbers for vascular density and max epithelial thickness are total counts, while the other criteria are a relative severity score ranging from 1 to 5.

### HoxA3 immunohistochemistry

Slides were deparaffinized with two 5-min incubation of histoclear (xylene substitute) followed by two changes of 100% ethanol, one change of 95% ethanol, one change of 70% ethanol, one change of 50% ethanol for 5 min each. Slides were then rehydrated with DI water for 5 min. Slides were then subject to antigen retrieval by submerging in pre-heated sodium citrate buffer (pH 6.0) in a staining dish for 30 min under heat and pressure cooker. After antigen retrieval, staining dishes with slides were removed from crockpot and allowed to cool on the benchtop for 30 min. Slides were permeabilized with three changes of 0.05% Tween-20 in PBS for 5 min each. Endogenous peroxidases were blocked by incubating slides in 3% hydrogen peroxide in PBS for 15 min. Non-target proteins were blocked by incubating slides in blocking buffer (5% BSA in PBS) for 2 h at room temperature. Primary antibodies for the human HoxA3 C terminus (ab230879, ABCAM) were diluted in blocking buffer and applied to the tissues. Slides were placed in a humidified chamber overnight at 4 °C. Primary antibody was removed with 3× washes of PBS. Biotinylated Goat Anti-Polyvalent (ab64256, ABCAM) secondary antibody was then applied to slides and allowed to incubate for 10 min at room temperature, followed by 3 washes of PBS. Streptavidin Peroxidase (ab64269, ABCAM) was then applied to slides and allowed to incubate for 10 min at room temperature, followed by 3 washes of PBS. DAB working solution was then applied to slides and allowed to incubate for 1.5 min. Slides were washed with PBS for 1 min each. Slides were counterstained with Hematoxylin for 2 min followed by a 1 min incubation in Bluing Reagent. Slides were dehydrated with one change of DI water, one change of 50% EtOH, one change of 70% EtOH, one change of 95% EtOH, and followed by two changes of 100% EtOH and two changes of Histoclear for 5 min each. Slides were mounted with Toluene Permount Solution and coverslips. For each tissue, 7 images were collected with a 40× objective. Images were masked in Image J for DAB positive nuclei and total nuclei per field of view. Percent HOXA3 positive nuclei was calculated by dividing DAB positive nuclei, by the total nuclei in the field of view.

### Statistics and data

A metric of “Average Wound Diameter (AWD) per measurement” was computed for each wound. This metric is essentially equivalent to “Area Under the Curve (AUC)” over the course of a wound closure trajectory. Average Wound Diameter is presented rather than AUC because AWD ranges between 0 mm (healed) and 6 mm (original hole size) and is more intuitive. AUC and AWD are numerically equivalent within a figure as they differ only in that AWD is divided by the number of observations which is constant and consistent across cohorts. AWD should not be directly compared between figures with differing observation frequencies. The underlying trajectory of measurements are shown, and observation days during which cohorts differed by statistically significant amounts of diameter (i.e. closure) were marked; generally studies were powered to achieve statistically significant AWD rather than to be statistically significant during individual days of observation.

**Figure 1:**
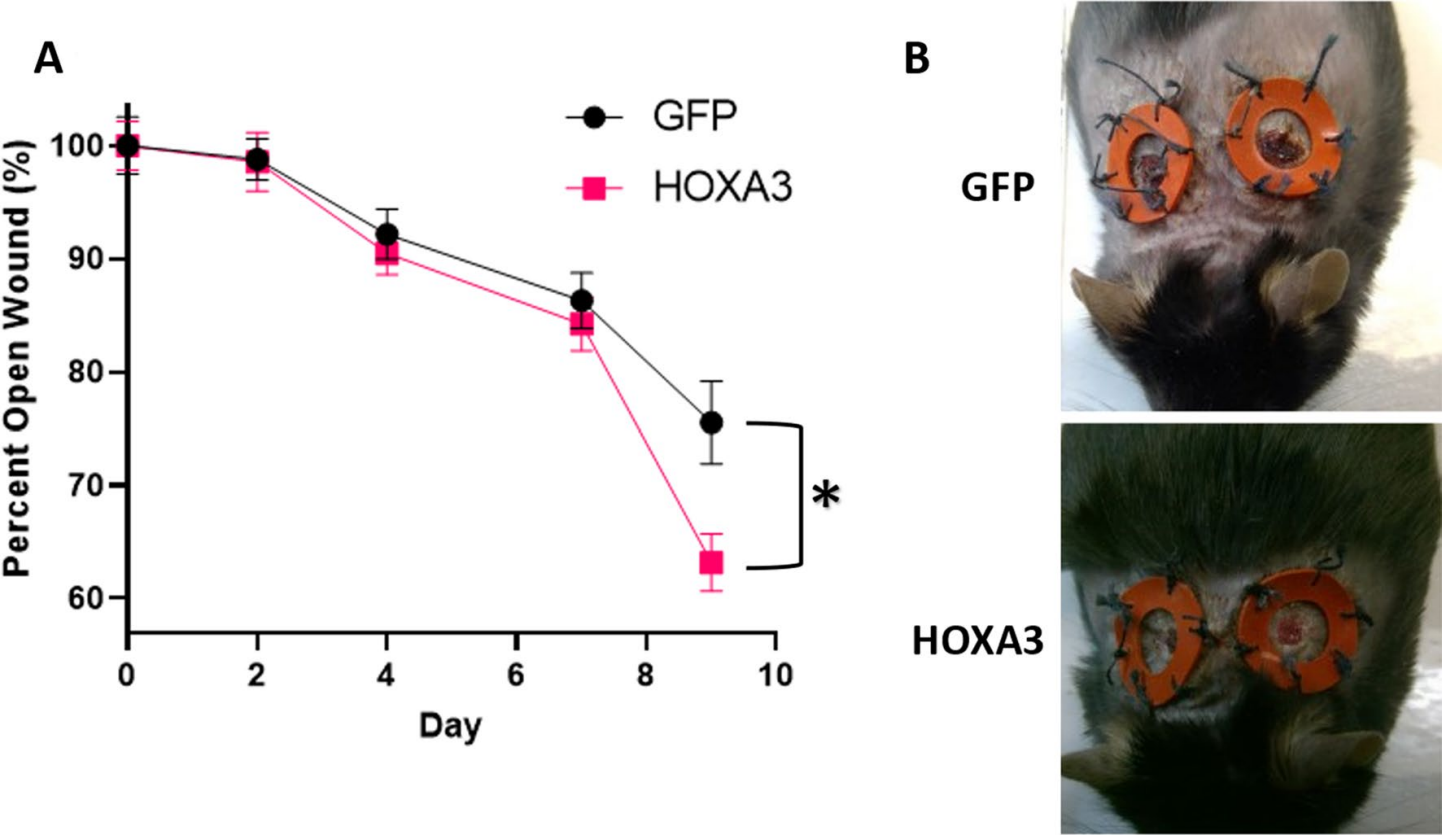
HOXA3 accelerates closure of diabetic wounds. (**A**) Mice received two wounds, left and right. GFP control mice (n = 9 animals because 1 animal died) and HoxA3 treated mice (n = 10) were measured at several timepoints over 9 days. Left and right wounds for each animal for each observation were averaged (adopting the assumption that animals are independent but wounds on an animal are not independent). At Day 9, the GFP treated average wound size was measured as 3.9 mm (76% of original wound size), while the HoxA3 treated wounds were recorded 3.3 mm (63% of original). Error bars are S.E.M. where each animal (not each wound) is considered independent. On Day 9, the HoxA3 treatment versus control values were separated by p < 0.02 by one-tailed heteroscedastic T-test and thus marked as statistically significant (*). Notably p < 0.04 assuming two-tail heteroscedastic T-test and, similarly, p < 0.04 when utilizing a Mann Whitney U one-tail test. (**B**) Representative images of wounds 9 days after wound induction. HoxA3 mice were generally re-epithelized giving the appearance of clearer skin vs the scabbed control.

**Figure 2:**
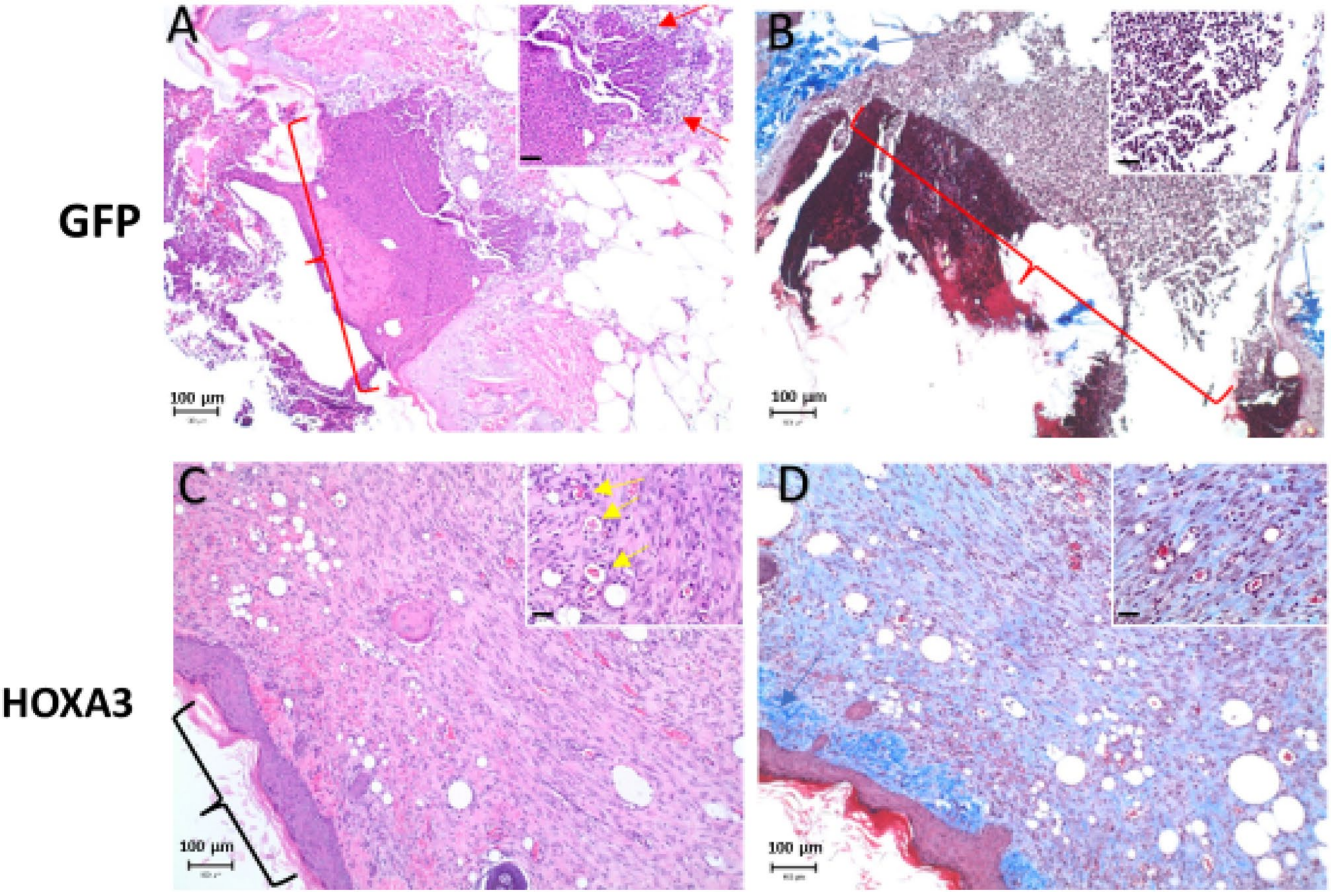
Representative histology in 9-day study of HoxA3 plasmid patches in diabetic mouse wounds. Untreated animals (**A**,**B**) have open wounds. At ×100 magnification in the HE (**A**,**C**) and trichrome (**B**,**D**) stained images, wound edges can be observed but there is an open area of wound in the center of the field and this is highlighted with a red bracket. This area is infiltrated by large number of inflammatory cells, predominantly neutrophils, which can be seen at ×400 magnification (inset, highlighted by red arrow). There is no notable granulation tissue with no observable new blood vessels nor fibrous tissue. Normal dermal collagen can be seen on the wound edge (blue arrow). Treated animals (**C**,**D**) have closed wounds with significant granulation tissue. At ×100 magnification the wound bed is covered by stratified squamous epithelium (black bracket) and there is marked granulation tissue underlying it. This tissue is composed of new blood vessels (yellow arrows) and fibrous tissue. The trichrome stained section highlights the fibrous tissue composing the granulation tissue. This new fibrous tissue is less densely staining and a lighter blue than the normal dermal tissue seen at the far left of the image (blue arrow). Scale bars in the larger images represent 100 µm, and the scale bars in the smaller images represent 25 µm.

Each animal received two wounds (e.g. left and right). Left and right wounds were found to be modestly non-independent. For each cohort, Pearson correlation coefficient was computed and ranged from − 0.35 to 0.93, with the simple average across cohorts being 0.42 ± 0.09. Pearson correlation coefficients of − 1.0 indicate perfect anti-correlation, 0.0 represents no correlation (wounds on the same animal are independent), and 1.0 represents total correlation (wounds on the same animal are totally dependent). A Pearson correlation coefficient of 0.42 corresponds to a squared value (i.e. R^2^) of 0.18, suggesting that the animal-specific characteristics explains 18% of the animal’s wound closure rate variance, with the other 82% contributed by truly independent wound-level recovery. Correlation of 0.42 can be interpreted as wounds on the same animal being moderately independent rather than completely dependent or independent, so the primary figures average each animal’s left and right wound observation rather than considering them as separate and each animal contributes itself to the sample size rather than its two wounds—this assumption is considered highly conservative. For studies where n = 5, the non-parametric Mann Whitney U test, with one-tail, is reported to determine statistical significance (p < 0.05). Where n = 9 or 10, the one-tailed heteroscedastic student’s T-test is used. One-tailed tests were utilized because of the a priori assumption that greater dosages would be superior. Alternative assumptions such as two-tailed p values are also mentioned in the text when relevant to statistical significance, and various permutations of statistical assumptions (parametric vs nonparametric, homoscedasticity vs heteroscedasticity, one vs two tails) are provided with the raw data in the Supplement. Compensation for multiple hypothesis testing (which applies in the dose dependence figure, where up to 4 dosages compared to control are made) was not done explicitly as highly conservative assumptions were already chosen, leaving the analysis underpowered, but it could be applied in combination with various other statistical assumptions made in the Supplement (Supplemental Information files 2, 3, 4, 5, and 6.xlsx corresponding to data for Figs. 1, 2, 4A,B, 5 respectively).

## Results

### Plasmids are expressed and bioactive

To confirm that the naked plasmids for HoxA3 and GFP are imbibed and expressed when placed in contact with cells, human Dermal Microvascular Endothelial Cells (hDMECs) were cultured with HoxA3 plasmid were evaluated for expression after 48 h incubation by immunocytochemistry via a primary antibody for the c-myc tag in the plasmid sequence and Cy2-conjugated secondary antibody. Similarly, hDMECs cultured with the GFP plasmid were evaluated for fluorescence directly. Fluoresence detection in HoxA3 treated and stained cells, as well as GFP treated cells, was clearly detectable versus untreated or unstained controls (Supplemental Figure S3).

Furthermore, hDMECs cultured into a monolayer and exposed to HoxA3 or GFP plasmid were evaluated for HoxA3 impact on motility. A scratch defect in the monolayer was inflicted and hDMEC infiltration into the denuded space was monitored. While cells in both treatment and GFP control groups successfully repopulated the gap within 40 h, HoxA3-treated hDMECs closed the defect more quickly than GFP-treated cells. These results demonstrate bioactivity of the plasmid upon contact with a relevant cell type (Supplemental Figure S4).

### Topical HOXA3 plasmid accelerates wound healing in diabetic mice at the microscopic levels

Diabetic mice (db/db) were randomized during the acclimation period for punch wounding and subsequent wound size monitoring until Day 10 post-injury, when mice were humanely sacrificed. One animal died during the acclimation period, leaving Group 1 (control) with 9 animals and Group 2 (treatment) with 10 animals. Treatment consisted of GFP (control, chosen due to its comparable molecular weight to HoxA3) or HOXA3 plasmid wafers (25 μg DNA plasmid in 1% methylcellulose, applied as 50 μL patches after 5 h of drying on waxed paper) applied to the wounds on Days 0, 2, 4, 7, and 9. Splints and sutures were replaced as needed throughout the study.

The primary intended outcome was histologic, but each animal in each group had wounds measured by digital calipers on days 0, 2, 4, 7 and 9 (Fig. 1). All animals were weighed on Days 0, 2, 4, 7, and 9. On Day 10, each group was humanely euthanized and wound areas were collected. From each animal, one wound was fixed in 10% neutral buffered formalin, while the other was flash frozen. Fixed wounds were embedded into paraffin blocks for microscopy and blinded pathologist scoring.

At Day 9, the final recorded data point before sacrifice, the HoxA3 cohort rapidly closed from roughly 85% open to approximately 63% open, whereas the control group remained 76% open. Although Day 9 was the first day of statistical significance, this fact was incidental, as the results were not tabulated until after sacrifice and Day 10 was merely chosen as the halfway point of untreated healing based on prior pilot studies (not shown).

**Figure 3:**
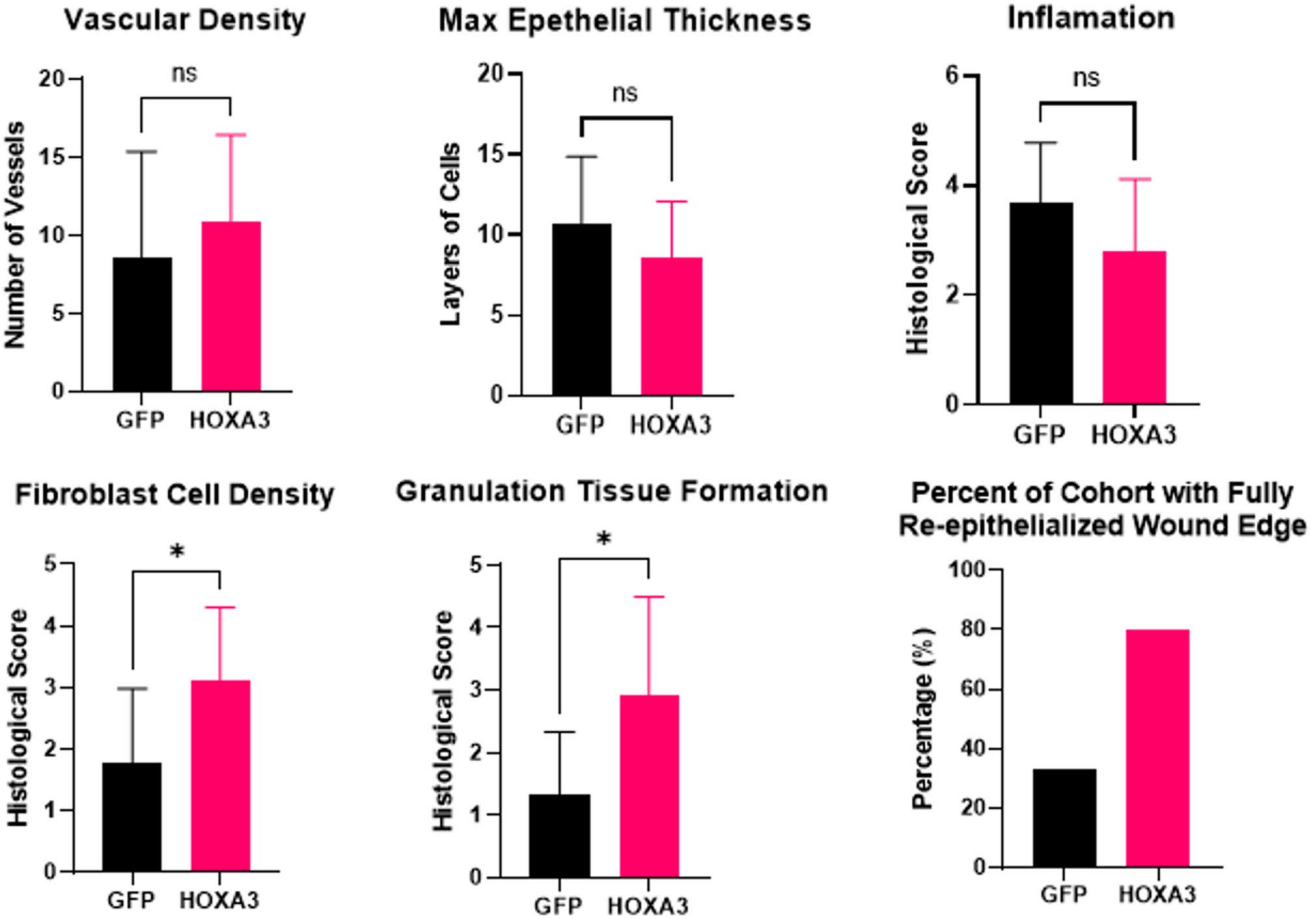
Histopathology scoring of 9-day diabetic wound healing fibroblast cell density, granulation tissue formation, and percent of cohort with fully epithelialized wound (which is a global count and thus has no error bars) were statistically significant (p < 0.05) in favor of HoxA3 when evaluated by two-tailed heteroscedastic Student’s T-test. Only one wound was evaluated per animal so left versus right statistical interpretation is not applicable.

Anonymized wounds were scored by a board-certified pathologist analyzing a 400× field from each wound. HoxA3 dramatically increased wound healing, granulation tissue formation, and fibroblast cell density. Nearly 80% of the HoxA3 wounds were fully re-epithelialized compared to only 20% for the controls (Fig. 3). Wounds treated with HoxA3 also had significantly more fibroblast cell density and granulation tissue formation than wounds treated with GFP (Figs. 2, 3). Figure 2D shows fibroblast cell density marked by the high presence of the extra-cellular matrix material. Vascular density was more pronounced in areas with granulation tissue. In Fig. 2C, the annotation marks patent blood vessels with red blood cells and tissue granulation in HoxA3 sample, which are absent in GFP-treated wounds. Due to within group animal to animal variation in overall healing, the increase in vascular density for HoxA3 treated animals did not reach statistical significance but was noticeably different (Fig. 3).

Inflammation was far more prominent in animals with open wounds than in those with largely healed wounds, as shown in in Fig. 2A compared to Fig. 2C. While HoxA3 treated wounds had reduced inflammation overall compared to GFP treated wounds, this change was not statistically significant (Fig. 3). Finally, the maximal epithelial thickness was measured. This was measured in only a single area of the slide showing the thickest skin and did not differ between groups. Additionally, as a check on plasmid expression, 7 images for each wound were collected and evaluated using algorithmically-defined image processing for HoxA3 nuclear expression by immunohistochemistry. While approximately 25% of untreated wound cells (generally keratinocytes) were positive for endogenous HoxA3 protein, total HoxA3 expression in treated wounds had over 45% HoxA3 positivity (Supplemental Figure S5).

**Figure 4:**
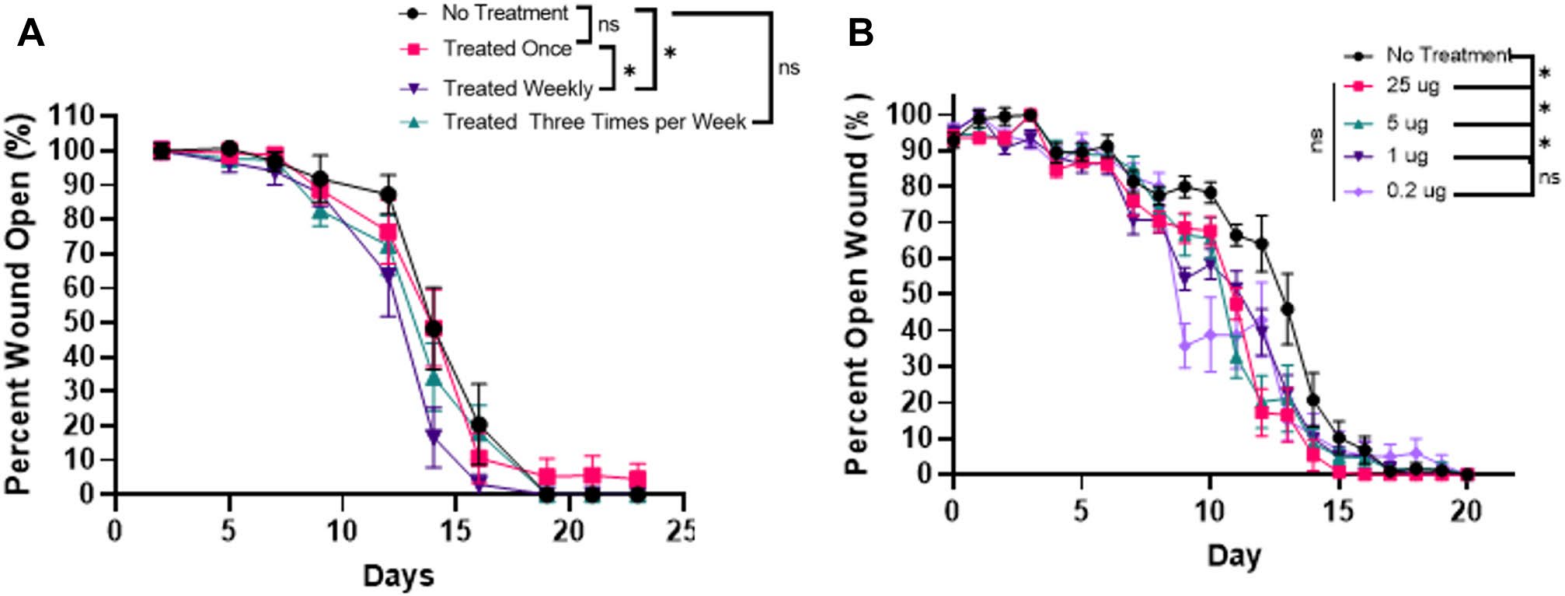
Diabetic wound closure HoxA3 (wt) dose dependency. (**A**) Various frequencies of treatment patch replacement were explored. Mice received two wounds, left and right. For negative control (untreated), 5 animals were injured and 4 survived (n = 4) whereas each animal in each HoxA3 cohort (n = 5) survived. (Top) left and right wounds for each animal in a cohort were first averaged, then S.E.M. error bars across animals were plotted. No individual days were statistically significant between controls and HoxA3 for any specific date under any of various statistical assumptions. The average wound diameter (connotated as AUC) was computed for each cohort. When comparing various HoxA3 frequency cohorts to untreated controls, Weekly-treated AUC was lower for weekly versus controls (and also versus Once-treatment) by 16%, with a p value < 0.04 by one-tail Mann–Whitney U test (p value < 0.08 for two-tail Mann Whitney U). ×3 weekly treatments were not statistically significant versus other cohorts and had an AUC that trended inferior to weekly but superior to GFP Control and weekly. (**B**) Various plasmid content dosages under weekly patch replacement were explored. Mice received two wounds, left and right (n = 5 animals per cohort). (Top) left and right wounds for each animal in a cohort were first averaged, then S.E.M. error bars across animals were plotted. No individual days were statistically significant between controls versus HoxA3 for any specific date under any of various statistical assumptions. (Bottom) the average wound diameter (connotated as AUC) was computed for each animal and plotted with error bars representing S.E.M. between animals in the cohort. Percentage benefit versus control was 12%, 12%, 19%, and 14% for 0.2 μg, 1 μg, 5 μg, and 25 μg, respectively. HoxA dosages of 1 μg, 5 μg, and 25 μg were statistically superior (p < 0.03) to GFP Control by one-tailed Mann Whitney U test, while 0.2 μg was not (p < 0.09). If two-tailed Mann Whitney U tests were used, then only 5 μg and 25 μg have p < 0.05 versus controls with p < 0.02 and p < 0.01, respectively.

A dose dilution study was conducted to determine the minimum plasmid dosing frequency and volume needed to accelerate healing. This minimum threshold was intended to be used to design experiments on improved therapies by determining the best dynamic range. First, mice were wounded and treated with 25 μg plasmid patches and monitored every 2 days for wound size by digital calipers. Mice received either three total doses, a single dose, or a non-treated control. Treatments were applied once at the time of wound induction, and once weekly every week for the duration of the study (i.e. 3 changes total), or 3× weekly (i.e. 9 changes total). Three total dosages (i.e. weekly) was effective while the control, single dose, and 3× weekly dosage plans were not (Fig. 4A). Technicians reported that each change appeared to damage the scab, which may have suppressed wound closure rates proportionate to the number of changes.

The impact of dosage quantity was then evaluated by varying plasmid content in the gel patches under the weekly (i.e. three-dose total) regime, as this was the most effective from the variable-frequency study. To improve temporal resolution, observations were made daily. Cohorts included either No Treatment, 25 μg wafer (akin to the most effective dose frequency approach), and intermediate dosages including 0.2 μg, 1 μg, and 5 μg. As shown in Fig. 4B, the 25 μg was nearly completely healed by Day 15 unlike the others.

### HoxA3 topical plasmid patch accelerates wound healing in non-diabetic aged mice

Aging is associated with the phenomenon “inflammaging”, as the immune system in older mammals enters an increasingly chronic state of low-grade immune activation with increasing age. Wounds are also known to heal more slowly in elderly human patients than in youth^35^. This effect is known to be partially macrophage mediated and is exacerbated in diabetic patients^36^. In a pilot study (Supplemental Figure S6) assessing the relative wound healing rates of aged wild-type mice (18 months of age), young wild-type and young diabetic mice (12 weeks of age), young wild-type mice healed quickly, requiring approximately 1 week. Whereas, both young db/db and old wildtype mice were similarly only 20% healed by Day 7. This similarity sparked the possibility that aged nondiabetic mice may benefit from HoxA3 plasmid, as diabetic mice do.

**Figure 5:**
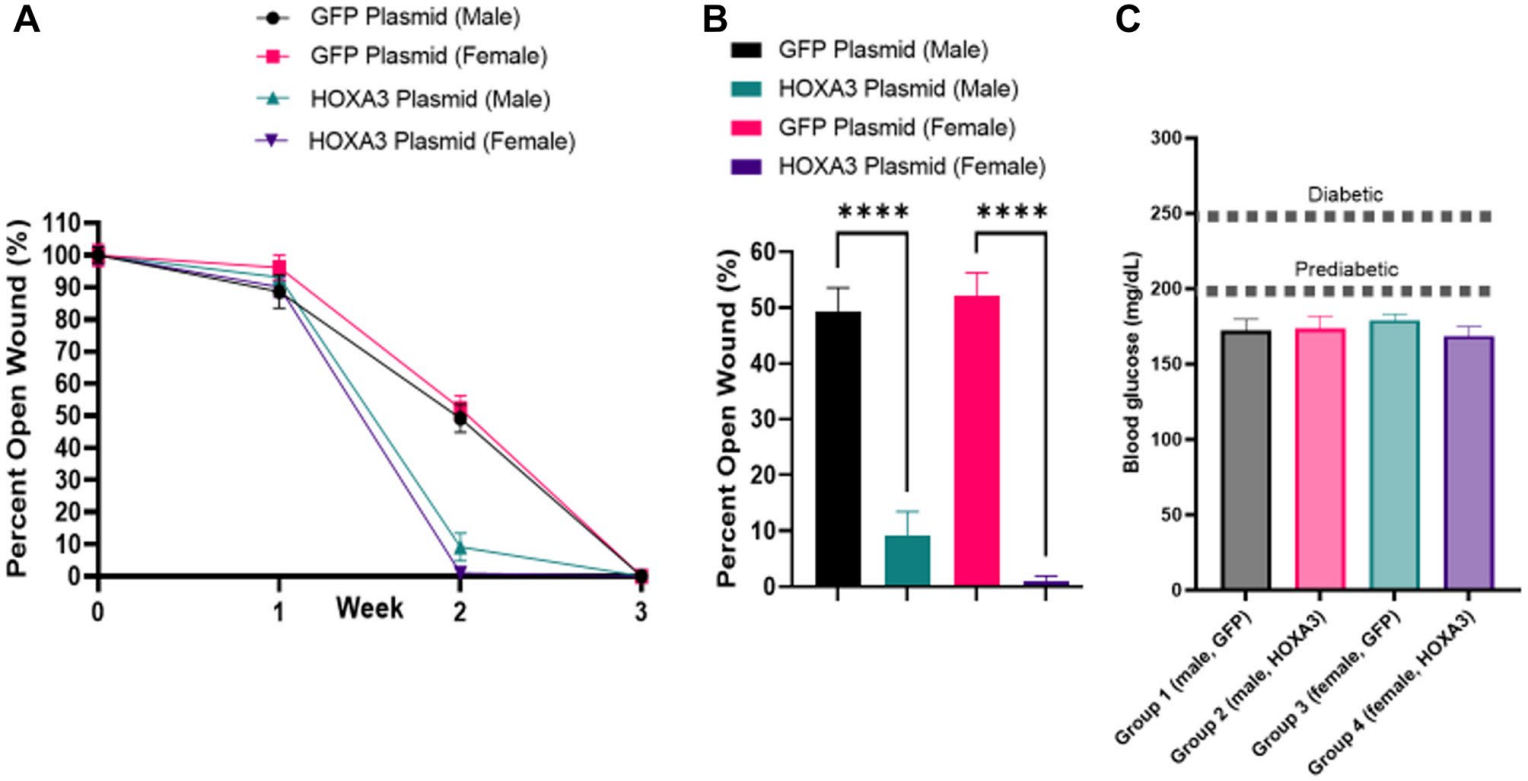
HoxA3 accelerates wound healing in aged, nondiabetic mice. Mice aged to 18 months then received two wounds, left and right, followed by weekly patch placement containing 25 μg plasmid (n = 9 per cohort, cohorts for male-control, male-HoxA3, female-control, female-HoxA3, one male-control died). (**A**) Wound size was measured weekly for each animal (averaging its left and right wounds for that day of observation) and plotted with error bars representing S.E.M. across animals. HoxA3 treated mice of both genders were mostly healed at 14 days post-injury while control mice of both genders were generally only 50% healed. For both genders, HoxA3 treated mice were statistically different from control mice on Day 14 regardless of statistical technique used (p < 10^–5^ by two-tail heteroscedastic T-test). (**B**) HoxA3 treated mice were statistically different from control mice on Day 14 regardless of statistical technique used (****p < 10^–5^ by two-tail heteroscedastic T-test). The average wound diameter at day 14 (connotated as AUC) was computed for each animal and plotted with error bars representing S.E.M. between animals in the cohort. Percentage benefit versus control was 13% in treated males and 22% in treated females versus their gender controls. AUCs were statistically significant in males and females versus their gender controls (p < 0.02) by two-tail heteroscedastic T-test, and indeed were p < 0.001 for females specifically. (**C**) Blood glucose measured at week 3 was ~ 180 mg/dL. For all panels, ns non-significant, with statistics determined by one-way ANNOVA. Data represent mean values; error bars represent S.E.M. Dotted lines represent established pre-diabetic and diabetic blood-glucose threshold^37^.

Given that aged wildtype mice appeared to respond to injury equivalently to diabetic young mice, for which HoxA3 has been shown to be effective, a formal study for HoxA3 was conducted on wildtype aged mice (Fig. 5). Mice were wounded as described previously and treated with 3 doses of 25 µg HoxA3 (treatment applied once at time of wound, and per week after). Both male and female mice were separately tested (n = 10). Mice were observed weekly by digital caliper for wound size. All wounds were closed at 21 days. No appreciable difference in wound closure at time of injury or at 1 week post injury was observed. However, at 2 weeks post injury, the treated wounds were consistently healed beyond 90% whereas the untreated wounds were consistently healed only approximately 50%. At 3 weeks, mice were sacrificed, and blood glucose was tested. No significant difference was found between any of the groups and all groups were nondiabetic^37^.

## Discussion

While the ability of HoxA3 plasmid (or paralogs like HoxD3^38^) to accelerate diabetic wound closure in vivo had been shown previously by the Mace and Boudreau labs, the similar effectiveness in non-diabetic aged mice is new. HoxA3 was shown previously in vitro to favor macrophage polarization to M2 gene expression, stimulate endothelial cell motility, and stimulate keratinocyte motility. Each of these factors are known to decrease during healthy aging in a process called “inflammaging”^35,36^. However, given that high blood glucose itself is known to be deleterious to wound healing via formation of Advanced Glycation Endproducts (AGEs) which are not directly targeted by HoxA3^39^, the effectiveness of HoxA3 in non-diabetic aging wounds is intriguing. Furthermore, the strong effect observed in both db/db and wildtype mice serves to alleviate concerns of strain specific effect.

Chronic wounds, both diabetic and nondiabetic, are a source of significant morbidity and cost affecting 1–2% of the general population in developed countries^40^. Among the elderly (age 65+), prevalence for chronic ulcers, pressure ulcers, and DFUs are 2.3%, 1.8%, and 0.7%, respectively^41^. The extremely elderly and ill patients are most at risk, and nursing home patients are particularly vulnerable to pressure wounds with a prevalence of 2.5%^42^. Despite the severity of this problem, no new treatments have been approved for chronic wounds since 1997 (becaplermin gel)^43^.

The wounds in this study were inflicted as sterile wounds to achieve controlled, homogenous injuries. While clinical reports vary, roughly 70% of chronic wounds are observed to have microbial biofilm presence^44^. Infections may need to be resolved prior to treatment with HoxA3, but this is untested. Because the wounds in this model were acute, and yet benefit was observed, the ideal application of HoxA3 may be as a home treatment for everyday wounds in the aged or elderly, or as an early intervention in pressure ulcers in a medical, long-term care, or assisted living facilities. Inflammaging is a cumulative process that becomes more noticeable in extreme old age but occurs continuously and is accelerated in patients with other health risk factors^45^; therefore, HoxA3 treatment might be most beneficial in elderly patients yet still useful in middle-age. The use of HoxA3 in inflamed wounds in youthful patients is more speculative, but stump wounds can become chronic due to abrasion with the prosthetic even without microbial involvement, and this environment is hyper-inflammatory^46^.

The work enclosed utilized a plasmid to achieve a durable effect. The Boudreau lab previously showed that the plasmids are not expressed in the peripheral blood at 4 days^29^. Plasmids generally do not integrate into the host genome and thus should be safe^47^. However, the regulatory path for naked plasmid-based therapies is sparsely populated in the US^48^. A protein may have an easier path to translation. While this study explored the minimal required dosing frequency and amount of plasmid, extrapolating the equivalent dose with HoxA3 topical protein is nontrivial both due to differences in degradation, cellular uptake, nuclear trafficking, and intracellular half-life and the harsh, protease-rich wound healing environment.

In theory, a topical treatment should minimize adverse effects versus systemic therapies. In this study, HoxA3 therapy was effective at 25 μg dosed once weekly, but not at a single dose in a 3-week study. However, a weekly dose was effective down to 1 μg. These results suggest that even modest dilution (3 or 5 fold, respectively) abrogates macroscopic biological effects which implies that nonlocal effects and thus adverse reactions of topical HoxA3 may prove manageable. Furthermore, while HoxA3 is pleitropic and affects many cell types (consistent with most Hox proteins), the immediate effects on the cells nearest to the application sites seem to be nonthreatening and potentially even beneficial. Inflammaging is generally considered deleterious, and macrophage repolarization would be a force of reversal. HoxA3 causes endothelial cells to become more motile and seems to promote angiogenesis, which is lacking in diabetic and aged tissue, although theoretically patients with active cancer should avoid angiogenic factors. Although we did not observe an increase in vascular density in the current study at the day 9 time point, previous work by Mace et al. observed increases in vascular density at earlier time points during the wound healing process. Increased keratinocyte motility would be undesirable in melanoma patients, but there is no early evidence of increased motility into unoccupied space corresponding to increased keratinocyte invasiveness. Many Hox genes are implicated in some form of cancer, in that constitutive expression seems to be a marker of worse survival, although some Hox genes seem to be protective in epithelial cancers, so the effects are likely cancer tissue type-specific and Hox-specific. HoxA3 in particular seems to have few associations to worsened cancer^49^.

In general, a plasmid or alternative modality for HoxA3 delivery appears to have significant translational potential. While plasmid uptake in relevant cell types has been shown in this and prior studies, bare Hox proteins can be modified for cellular uptake and activity^50^ and liquid nanoparticle encapsulation can preferentially target macrophages^51^. A clear path exists from most in-need patients (diabetic wound), to chronic wounds of the elderly in a medical care setting, to perhaps over-the-counter acute injury ointment or bandages, and potentially to wounds in younger adults with special inflammatory regions such as amputation stump wounds.

## Supplementary Information

Supplementary Figures.

Supplementary Information 1.

Supplementary Information 2.

Supplementary Information 3.

Supplementary Information 4.

Supplementary Information 5.

Supplementary Information 6.

Supplementary Information 7.

Supplementary Video 1.

Supplementary Video 2.

## Data Availability

All data generated or analyzed during this study are included in this published article (and its supplementary information files).
