# Supplementary material for: HOXA3 accelerates wound healing in diabetic and aged non-diabetic mammals
Source: Sci Rep. 2023 Jun 19;13:9923. doi: 10.1038/s41598-023-36933-4 (PMC10279746; doi:10.1038/s41598-023-36933-4)
Supplement: Supplementary file 1 — Supplementary Figures. [file 41598_2023_36933_MOESM1_ESM.docx]

**SUPPLEMENTAL INFORMATION:**


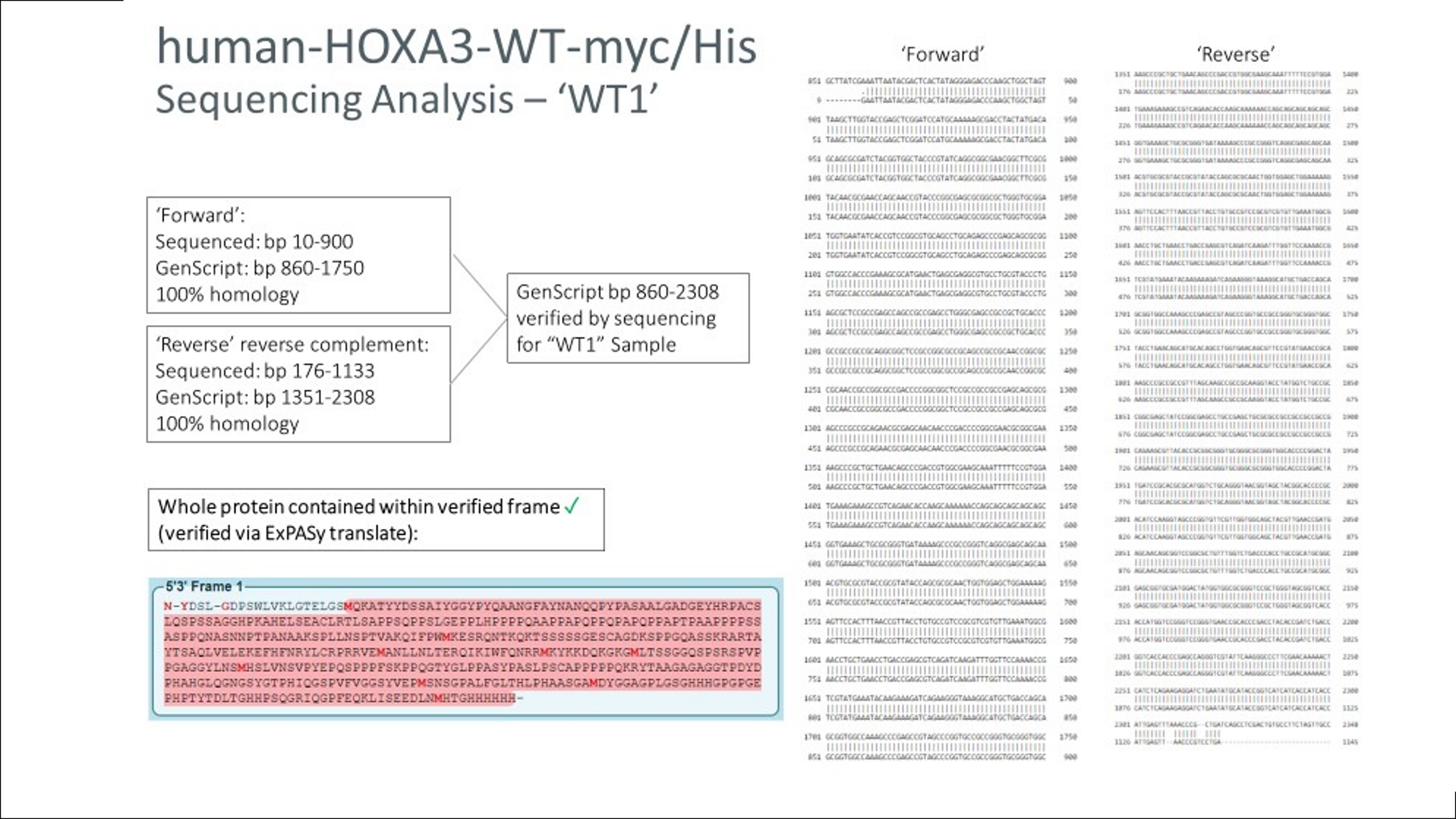


**Figure S1** The gene corresponding to human WT HOXA3 with c terminus myc and his tags was cloned into pcDNA3.1 vector. After the plasmid was replicated, sequencing was performed to ensure no mutations were introduced.


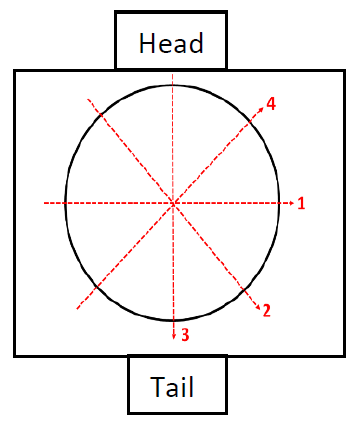


***Figure S2. Wound measurement policy.*** *Wounds were measured manually using digital calipers by taking 4 measurements and averaging. The first measurement was aligned head-to-tail and the subsequent measurements were aligned at 45 degrees to each previous measurement to obtain even coverage. The final average was recorded as the size for the given wound for the given day.*


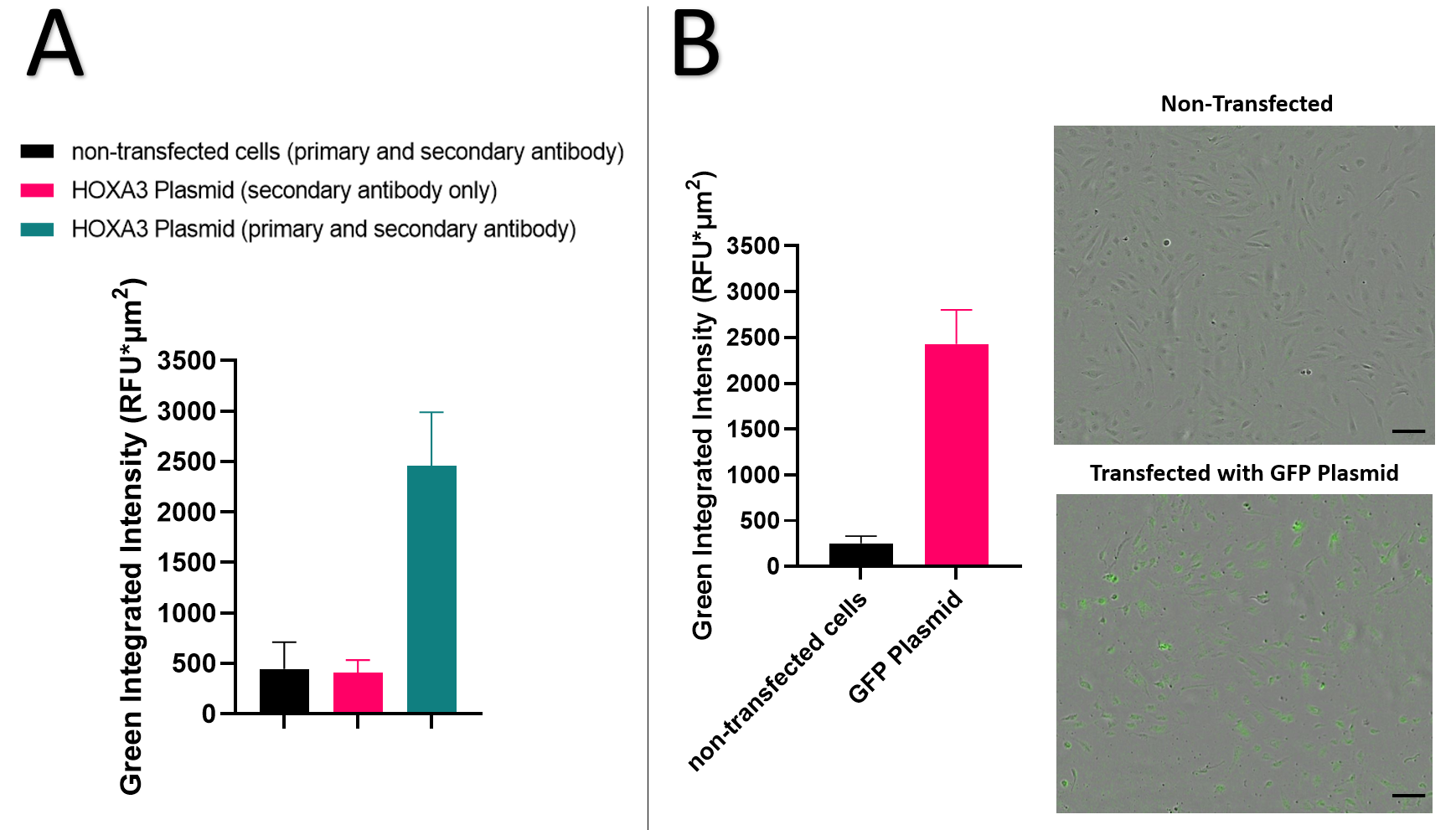


***Figure S3. Plasmid Expression in Cultured Human Dermal Microvascular Endothelial Cells (DMECs).*** *Human DMECs were transfected with either GFP plasmid or equivalent HoxA3 plasmid. After 48 hours incubation, (A) by immunocytochemistry for exogenous HoxA3 expression using a c-myc tag or (B) direct fluorescence of GFP plasmid. Plasmid payload expression was confirmed. Error bars represent S.E.M, n=4. Scale bars on images represent 100 µm*


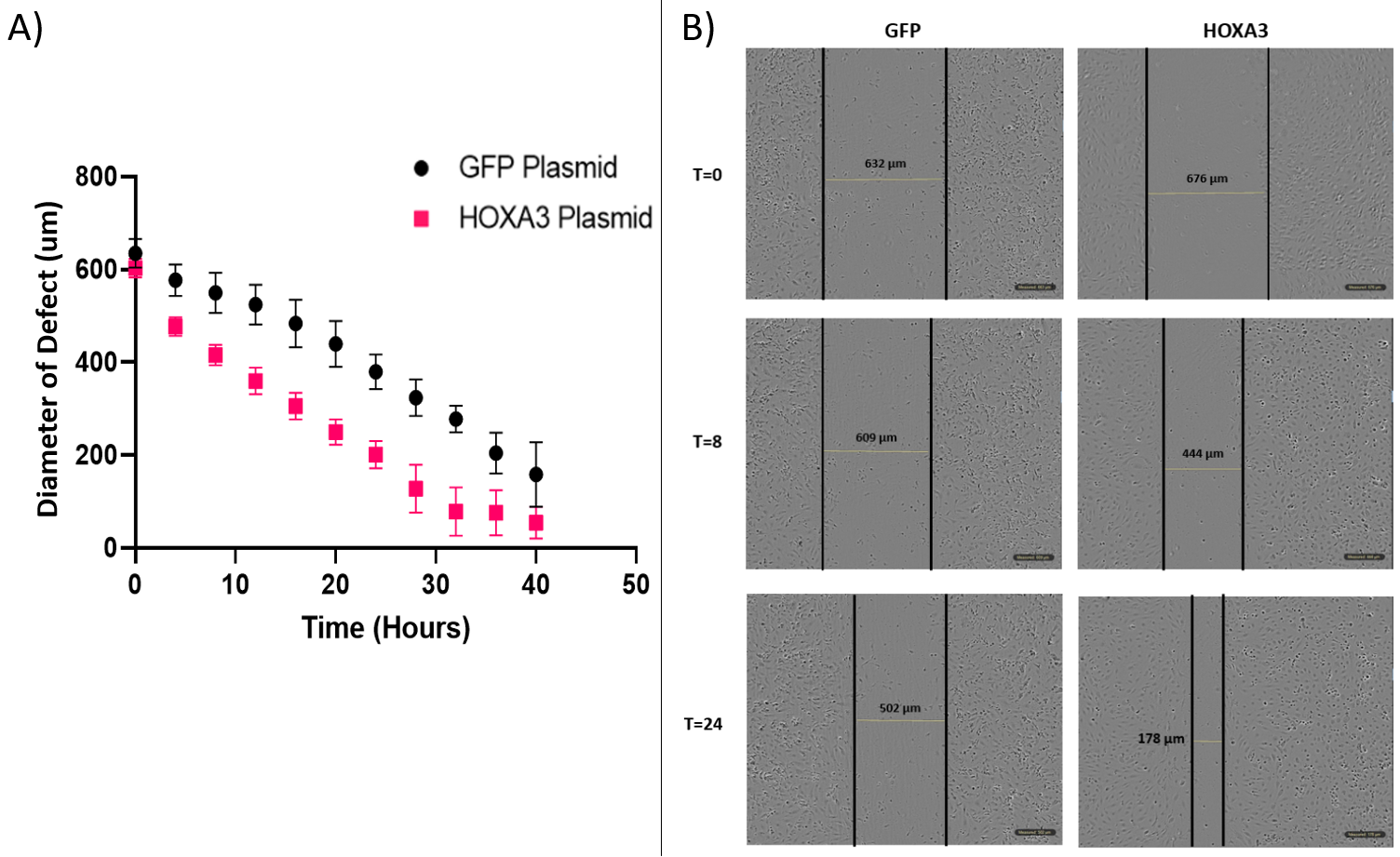


***Figure S4. Motility of hDMECs after Induced Plasmid Expression in Vitro.*** *Human DMECs were transfected with either GFP plasmid or equivalent HoxA3 plasmid. After 48 hours incubation, (A) by immunocytochemistry for exogenous HoxA3 expression using a c-myc tag or (B) direct fluorescence of GFP plasmid. Plasmid payload expression was confirmed. Error bars represent S.E.M, n=4. Scale bars on images represent 100 µm.*


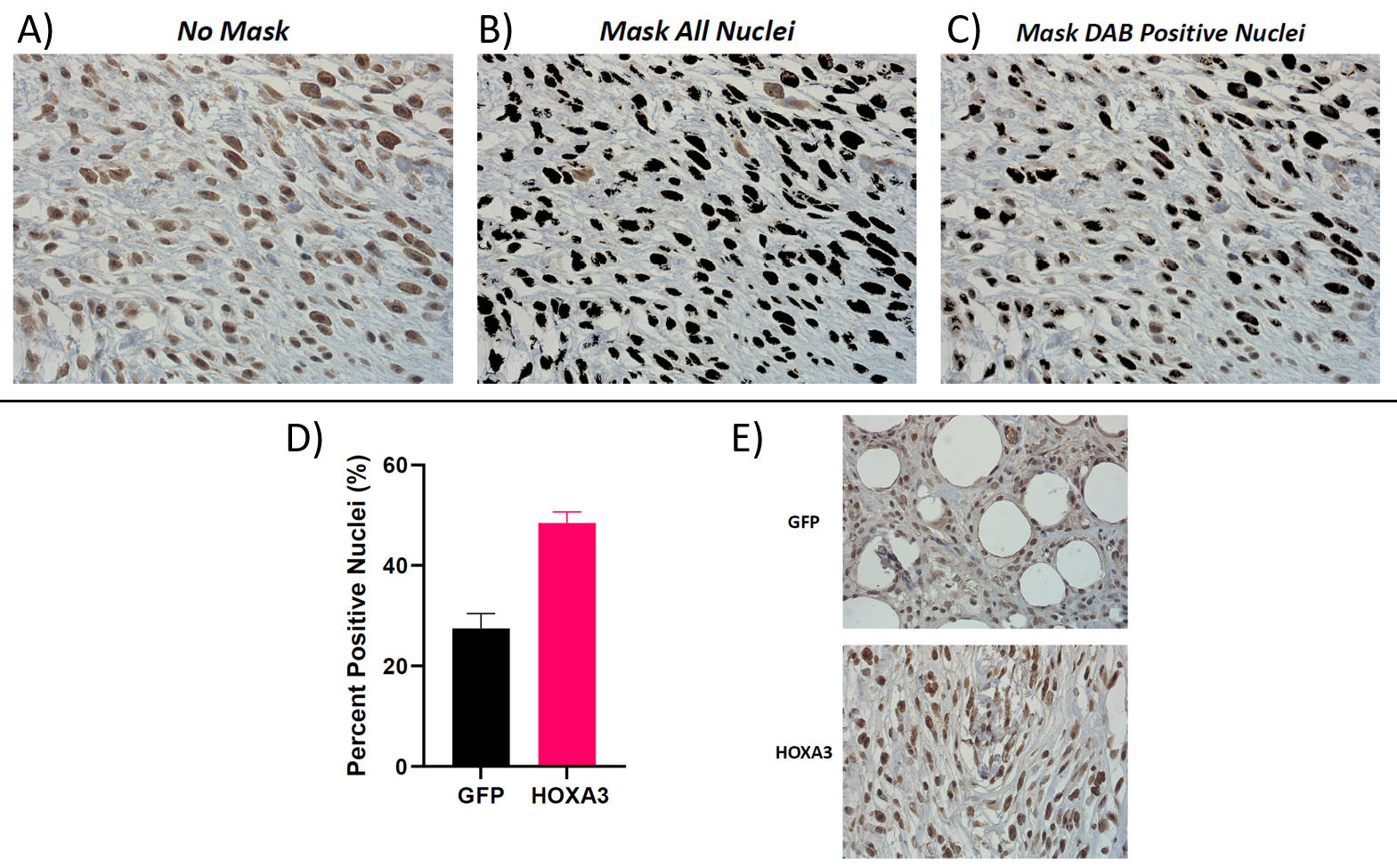


***Figure S5. Expression of HoxA3 in Treated Wounds.*** *(A) Excised wounds were mounted on slides at Day 9 post-injury. Slides were stained with primary antibody for HoxA3. (B) A programmatic masking algorithm was used to identify nuclei using native instrument functionality. (C) An programmatic masking algorithm was used to distinguish HoxA3 positive from HoxA3 negative nuclei. (D) 7 images per wound were quantified. Wounds from the HoxA3-treated animals displayed a higher fraction of HoxA3+ nuclei. (E) Example image of control (GFP) and treated (HoxA3) wound images.*

**
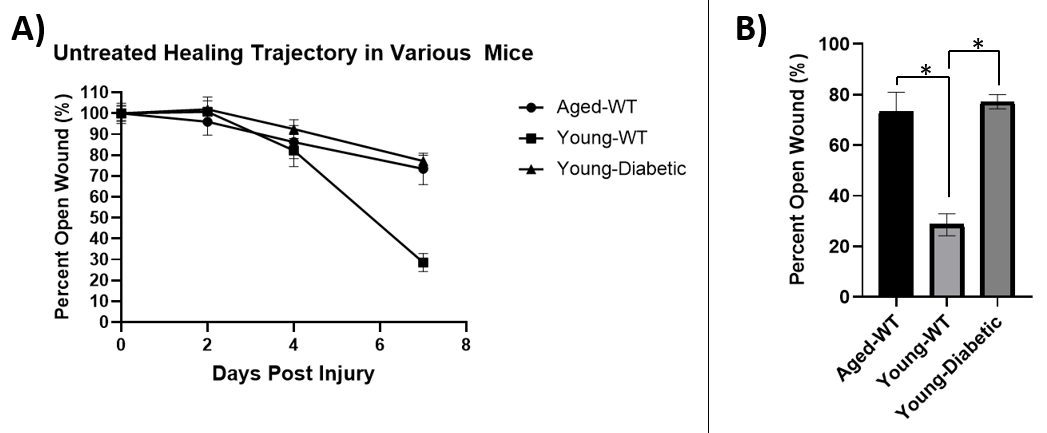
**

***Figure S6. Untreated Wound Healing Trajectories.*** *(A) Mice were wounded and observed for 7 days. Cohorts included aged wildtype mice (18 months), young wildtype mice 12 weeks or young db/db mice (12 weeks). Young mice recovered roughly 80% while aged and db/db mice recovered only about 20% by day 7*. *(B) column analysis of the final day of wound measurement. ns non-significant, * p< 0.0001, statistics determined by One-Way ANNOVA. Data represent mean values; error bars represent S.E.M*
